# Expression of oestrogen receptors, ERα, ERβ, and ERβ variants, in endometrial cancers and evidence that prostaglandin F may play a role in regulating expression of ERα

**DOI:** 10.1186/1471-2407-9-330

**Published:** 2009-09-16

**Authors:** Frances Collins, Sheila MacPherson, Pamela Brown, Vincent Bombail, Alistair RW Williams, Richard A Anderson, Henry N Jabbour, Philippa TK Saunders

**Affiliations:** 1 MRC Human Reproductive Sciences Unit, The University of Edinburgh Centre for Reproductive Biology, Queen's Medical Research Institute, 47 Little France Crescent, Edinburgh EH16 4TJ, UK; 2 Division of Reproductive and Developmental Sciences, The University of Edinburgh Centre for Reproductive Biology, Queen's Medical Research Institute, 47 Little France Crescent, Edinburgh EH16 4TJ, UK; 3 Division of Pathology, The University of Edinburgh, Simpson Centre for Reproductive Health, 51 Little France Crescent, Edinburgh EH16 4SA, UK

## Abstract

**Background:**

Endometrial cancer is the most common gynaecological malignancy; risk factors include exposure to oestrogens and high body mass index. Expression of enzymes involved in biosynthesis of oestrogens and prostaglandins (PG) is often higher in endometrial cancers when compared with levels detected in normal endometrium. Oestrogens bind one of two receptors (ERα and ERβ) encoded by separate genes. The full-length receptors function as ligand-activated transcription factors; splice variant isoforms of ERβ lacking a ligand-binding domain have also been described. PGs act in an autocrine or paracrine manner by binding to specific G-protein coupled receptors.

**Methods:**

We compared expression of ERs, progesterone receptor (PR) and cyclooxygenase-2 (COX-2) in stage 1 endometrial adenocarcinomas graded as well (G1), moderately (G2) or poorly (G3) differentiated (n ≥ 10 each group) using qRTPCR, single and double immunohistochemistry. We used endometrial adenocarcinoma cell lines to investigate the impact of PGF2α on expression of ERs and PR.

**Results:**

Full length ERβ (ERβ1) and two ERβ variants (ERβ2, ERβ5) were expressed in endometrial cancers regardless of grade and the proteins were immunolocalised to the nuclei of cells in both epithelial and stromal compartments. Immunoexpression of COX-2 was most intense in cells that were ERα^neg/low^. Expression of PR in endometrial adenocarcinoma (Ishikawa) cell lines and tissues broadly paralleled that of ERα. Treatment of adenocarcinoma cells with PGF2α reduced expression of ERα but had no impact on ERβ1. Cells incubated with PGF2α were unable to increase expression of PR mRNA when they were incubated with E2.

**Conclusion:**

We have demonstrated that ERβ5 protein is expressed in stage 1 endometrial adenocarcinomas. Expression of three ERβ variants, including the full-length protein is not grade-dependent and most cells in poorly differentiated cancers are ERβ^pos^/ERα^neg^. We found evidence of a link between COX-2, its product PGF2α, and expression of ERα and PR that sheds new light on the cross talk between steroid and PG signalling pathways in this disease.

## Background

Endometrial cancer is the most common gynaecological malignancy and accounts for 5% of cancers in women http://info.cancerresearchuk.org/cancerstats/. The majority of endometrial cancers occur in post-menopausal women and 80% of patients are diagnosed when the tumour is confined to the uterus (stage 1 disease). Many of the established risk factors for developing endometrial cancer are associated with excess exposure to oestrogen unopposed by progesterone. For example, several studies have reported that use of oestrogen-only hormone replacement therapy (HRT) increases the risk of developing both localized and widespread endometrial cancer [1,2]. The menopausal transition (perimenopause), a time when oestrogens may be elevated and anovulatory cycles mean that progesterone levels are reduced, has been proposed as a possible 'window of risk' for the development of the disease [3]. A high body mass index (BMI) [4,5] increases the risk of developing endometrial cancer and patients with a high BMI have a poorer prognosis [6]. Expression of enzymes involved in biosynthesis of oestrogens such as CYP19A1 and 17β HSD type 2 have been documented in endometrial carcinomas [7,8] and concentrations of oestradiol (E2) in tumour tissues have been correlated positively with the clinical stage of disease and rate of tumour invasion in both pre- and post-menopausal women [9].

The impact of oestrogenic ligands on endometrial cells is mediated via oestrogen receptors that act as ligand-activated transcription factors. There are two oestrogen receptors, ERα [*ESR1*] and ERβ [*ESR2*], encoded by different genes. The human ERβ gene is alternatively spliced at its 3' end resulting in formation of mRNAs that encode not only a full-length protein (ERβ1) capable of binding to E2 but also truncated isoforms (ERβ2, ERβ5) lacking an intact binding pocket [10]. Expression of ERs in normal pre-menopausal endometrium has been well documented with immunoexpression of ERα being intense in both glands and stroma during the proliferative, oestrogen-dominant phase but reduced in the secretory phase following the post ovulatory rise in progesterone [11]. ERβ1 and ERβ2 are both expressed during the proliferative phase however following ovulation ERβ1 continues to be expressed, ERβ2 is selectively down-regulated in the glandular epithelium [12] and the pattern of expression of ERβ5 has not been described.

In normal endometrium expression of progesterone receptor (PR) is induced during the oestrogen-dominated proliferative phase and a number of response elements capable of activation by ERs have been described within the regulatory region of the PR gene [13]. During the secretory phase when circulating concentrations of progesterone are maximal activation of PR results in reduced proliferation and increased cellular differentiation. If progesterone biosynthesis is inadequate/absent as might occur during anovulatory cycles the endometrium can become hyperplastic. Notably, development of complex atypical hyperplasia carries a 25% risk of developing subsequent endometrial adenocarcinoma. Biochemical studies record lower concentrations of ER and PR in endometrial cancers from clinical stages III-IV than those from clinical stage I; in stage I samples higher concentrations of receptor were measured in the well and moderately differentiated samples [14]. In endometrial carcinomas mRNAs for several ERβ isoforms have been detected [15-17] but detailed immunolocalisation studies comparing their expression have not been reported. It has been claimed that PR immunohistochemistry provides the most reliable means for predicting survival in endometrial adenocarcinoma [18], that detection of PR is associated with better disease free survival [19] and that administration of progestins is an effective treatment for pre-menopausal women with endometrial carcinomas or atypical hyperplasia [19].

In the reproductive tract, the predominant prostaglandins are the E- and F-series prostanoids [20]. These are synthesised from arachidonic acid by cyclooxygenase (COX) and prostaglandin synthase enzymes and act in an autocrine or paracrine manner by binding to specific G-protein coupled receptors (GPCR; reviewed in [21]). There is emerging evidence supporting a complex interplay between the production and action of oestrogens and prostaglandins within the microenvironment of tumours and endometrial pathologies such as endometriosis. For example, E2 can increase expression of COX enzymes [22,23] and the existence of an oestrogen response element has been documented in the promoter of the gene encoding prostaglandin synthase enzymes [24]. There is convincing evidence that PGE2 stimulates biosynthesis of oestrogens by enhancing expression of the aromatase (CYP19A1) gene in endometriotic tissue [25] and expression of aromatase can be suppressed by COX-2 selective inhibitors [26].

In endometrial adenocarcinoma, expression of COX-2 but not COX-1 is upregulated compared with normal endometrium [27,28]. Moreover, we have demonstrated a role for the F Prostanoid (FP) receptor (the receptor for prostaglandin PGF2α) in endometrial adenocarcinoma, with evidence that elevated PGF2α-FP receptor signalling results in an up regulation in expression of angiogenic and tumorigenic genes including COX-2 [29], FGF2 [30] and VEGF [31] as well as an increase in proliferation and migration of neoplastic epithelial cells [32]. In the present study we investigated whether expression of ERs, including ERβ variants, could be correlated with the degree of differentiation of grade 1 tumours and/or expression of PR and COX-2. We also investigated the impact of PGF2α on expression of ERα, ERβ and PR in cancer-derived endometrial epithelial cells.

## Methods

### Patients and tissue collection

Endometrial adenocarcinoma tissue was collected from post-menopausal women undergoing total abdominal hysterectomy who had been previously diagnosed to have endometrioid adenocarcinoma of the endometrium; they had received no treatment before surgery. Written informed consent was obtained from all patients; ethical approval was obtained from the Lothian Research Ethics Committee. All endometrial cancers were confined to the uterus (International Federation of Obstetrics and Gynaecology, FIGO, stage 1 [33]). Diagnosis of adenocarcinoma was confirmed histologically and tissues were further graded as well differentiated (G1), moderately differentiated (G2) or poorly differentiated (G3) by an experienced gynaecological pathologist. A minimum of 10 samples at each grade was analysed, tissue for immunohistochemistry was collected in neutral buffered formalin (NBF) RNA extraction samples were collected in RNALater (Qiagen, UK).

### Cell cultures

Two endometrial adenocarinoma cell lines derived from different patients were used. The first cell line [Ishikawa A] was obtained from the European Collection of Cell Culture (ECACC no 99040201, Wiltshire, UK) and maintained in DMEM (Sigma, Poole, UK). This cell line was originally derived from a well-differentiated adenocarcinoma of a 39 year-old woman and characterised as containing ER and PR [34]. A second cell line [Ishikawa B], previously characterised as ERα-negative [35], was derived from the tumour of an unrelated patient with the same last name. Cells were maintained in DMEM (Sigma) supplemented with 10% FBS, 100 U penicillin, streptomycin and 0.25 ug/ml fungizone (Invitrogen, Paisley, UK) at 37°C in 5% CO_2_. An additional cell line derived from the Ishikawa A cells following stable transfection with FP receptor cDNA [ERα^pos^/FP^pos^] was maintained with the addition of 200 μg/ml G418 [31]. Cells were treated with oestradiol 17β (E2) using stocks diluted in DMSO to give final concentrations in the range 10^-7 ^to 10^-10 ^M or prostaglandin F2α at a final concentration of 100 nm [stock solution prepared in ethanol]; appropriate vehicle control incubations were included in all studies.

### RNA extraction and Taqman quantitative RT-PCR

Total RNA was extracted using the RNAeasy mini kit (Qiagen, Sussex, UK) with additional purification by centrifugation through QIAshredder spin columns (Qiagen). RNA concentration and purity was calculated using the Nanodrop (LabTech International, Lewes, Sussex, UK) and standardised to 100 ng/μl for all samples. The reverse transcriptase reaction consisted of 400 ng of RNA, 2.5 μM random hexamers in 1× PCR buffer II, 5 mM MgCl_2_, 1 mM dNTP's, 1 U/μl RNase inhibitor and 2.5 U/μl Multiscribe RT (Applied Biosystems, Foster City, USA) incubated at 25°C for 20 minutes, 42°C for 60 minutes followed by 5 minutes at 95°C. A pooled RNA control supplied by ABI was included as a reference sample in all reactions. Quantitative PCR was performed using FAM labelled probes from the Universal Probe Library (Roche Diagnostics, Burgess Hill, UK) and specific primers for the ERα, ERβ1, 2, 5 and PR (Table 1). Each 20 μl reaction consisted of 2 μl of cDNA in 1× Faststart master mix (Roche) with additional Rox dye to a final concentration of 510 nM with 200 nM of forward and reverse primer, 50 nM probe, 0.02 μM of 18S primers and 0.08 uM 18S probe; 40 cycles of PCR [95°C for 15 s followed by 60°C for 1 minutes] were carried out using the ABI Prism 7900HT sequence detection system (Applied Biosystems, Foster City, USA).

**Table 1:** Details of primers and probes used for quantitative PCR

cDNA	Forward Primer	Reverse Primer	Roche Probe
ERα	ttactgaccaacctggcaga	atcatggagggtcaaatcca	24
ERβ1	gctcctgtcccacgtcag	tgggcattcagcatctcc	62
ERβ2	tgggtgattgccaagagc	gtttgagaggccttttctgc	52
ERβ5	gctcctgtcccacgtcag	cacataatcccatcccaagc	17
PR	tttaagagggcaatggaagg	cggattttatcaacgatgcag	11

### Luciferase ERE reporter assays

An adenoviral vector containing a 3xERE-tk-luciferase reporter gene was prepared according to a standard protocol (Microbix) from a plasmid that was a kind gift from Professor DP McDonnell ([36], Duke University NC, USA). A full-length human ERα cDNA (see [37]) was used to prepare viral constructs using an identical strategy. The resulting viral particles were plaque purified, amplified in Hek293 cells and concentrated using a commercial kit (Vivascience). Cells [1 × 10^5^] were plated in 24 well tissue culture plates in DMEM containing charcoal stripped foetal calf serum (CSFCS) and cultured for 24 hours before being infected with Ad-ERE-Luc at a MOI of 100. After a further 24 hours cells were incubated with E2 [10^-7 ^to 10^-10 ^M] for 24 hours and luciferase activities were determined using 'Bright Glo' reagents (Promega,).

### Immunohistochemistry

#### Single antibody immunohistochemistry (IHC)

Details of primary antibodies are given in Table 2. The specificity of the antibodies directed against the ERβ variants has already been validated using Western blotting [38,39]. Slide-mounted 5 μm sections were subjected to heat-induced antigen retrieval according to standard methods [40] (Table 2). Sections were incubated with 3% (v/v) hydrogen peroxide in methanol for 30 minutes to block endogenous peroxidase, washed and transferred into Tris-buffered saline (TBS; 0.05 M Tris [pH 7.4], 0.85% saline) for 5 minutes. Non-specific binding was blocked using normal rabbit serum (NRS, Biosera) diluted 1:4 in TBS containing 5% BSA (NRS/TBS/BSA) [ERα, ERβ2] or normal goat serum (NGS, Biosera) diluted 1:4 in TBS containing 5% BSA (NGS/TBS/BSA) [ERβ1, ERβ5]. An avidin biotin block was performed using reagents from Vector (Blocking kit, Cat. No. SP-2002, Peterborough, UK). Primary antibodies were diluted in the appropriate blocking serum (Table 2) and incubated on sections overnight at 4°C. Sections were washed twice and incubated with the appropriate biotinylated secondary antibodies diluted at 1:500 (30 min), washed again and incubated in Streptavidin-HRP (DAKO; P0397) for 30 minutes, before bound antibodies were visualized by incubation with 3,3'-diaminobenzidine tetra-hydrochloride (liquid DAB+, product no. K346811, Dako).

**Table 2:** Details of primary antibodies

Target		Clone	Supplier	DAB concentration	Antigen retrieval
ERα	mouse	6F-11	Novocastra	1:120	0.01 M Citrate buffer (pH6) for 5 minutes
ERβ1	mouse	PPG5/10	Serotec, [60]	1:40	0.05 M Glycine/EDTA (pH8) for 5 minutes
ERβ2	mouse	57/3	Serotec, [38]	1:30	0.05 M Glycine/EDTA (pH8) for 5 minutes
ERβ5	mouse	5/25	Serotec, [61]	1:75	0.01 M Citrate buffer (pH6) for 5 minutes
PR (A+B)	rabbit	C19	Santa Cruz	N/A	0.01 M Citrate (pH6) 10 min
COX-2	goat	C20	Santa Cruz	N/A	0.01 M Citrate (pH6) 10 min

**Details of secondary antibodies are given in Table 4.

#### Double fluorescent immunohistochemistry

An overview of the protocol used for each of the combinations of antibodies used for double fluorescent immunohistochemistry is summarised in Table 3. Note the antibody directed against PR will cross-react with both A and B forms of the protein. In all cases initial antigen retrieval was carried out in citrate buffer [40], biotin conjugates were diluted in NGS/PBS/BSA, fluorescent conjugates were diluted in PBS, primary antibodies were diluted in NGS/PBS/BSA and incubated overnight at 4°C. All washes carried out between antibody incubations were in PBS and were repeated twice for 5 minutes each. Details of secondary antibodies and stains are given in Table 4.

**Table 3:** Details of protocols used for fluorescent co-localisation.

ERβ1+PR	ERα +PR	COX2+ ERα
Citrate retrieve	Citrate retrieve	Citrate retrieve
Methanol/peroxide block	NGS block	NRS block
NGS block	Avidin block	Avidin block
Avidin block	Biotin block	Biotin block
Biotin block	ERα 1:20	COX-2 1:60
PR 1:50	GAM 488	RAGB
GARB	NGS block	Streptavidin 546
Streptavidin 546	PR 1:50	NGS
NGS block	GARB	ERα 1:20
ERβ1 1:200	Streptavidin 546	GAM 488
GAMP	To-Pro	To-Pro
Tyramide fluoresein		
To-Pro		

The protocol for each combination of stains is given in the vertical columns; all sections are pre-blocked with normal goat serum

**Table 4:** Secondary antibodies and counterstains

Antibody	Abbreviation	Supplier	**Product no**.	Dilution
Goat anti rabbit biotinylated	GARB	Dako	E0432	1:500
Goat anti mouse biotinylated	GAMB	Dako	E0433	1:500
Goat anti-mouse Alexa Fluor 488	GAM 488	Mol. Probes	A-11029	1:200
Avidin Alexa Fluor 488	Avidin 488	Mol. Probes	A-21370	1:200
Streptavidin Alexa Fluor 546	Streptavidin 546	Mol. Probes	S-11225	1:200
Tyramide fluorescein	Tyramide fluorescein	Perkin Elmer Life Sciences	NEL 744	1:50
To Pro	To Pro	Mol. Probes	T3605	1:1000

### Statistical analysis

Statistical differences were determined by ANOVA followed by post hoc Bonferroni multiple comparison test. Values are expressed as mean +/- SD and P < 0.05 was considered statistically significant.

## Results

### Expression of oestrogen receptors in stage 1 endometrial cancers

**Figure 1:**
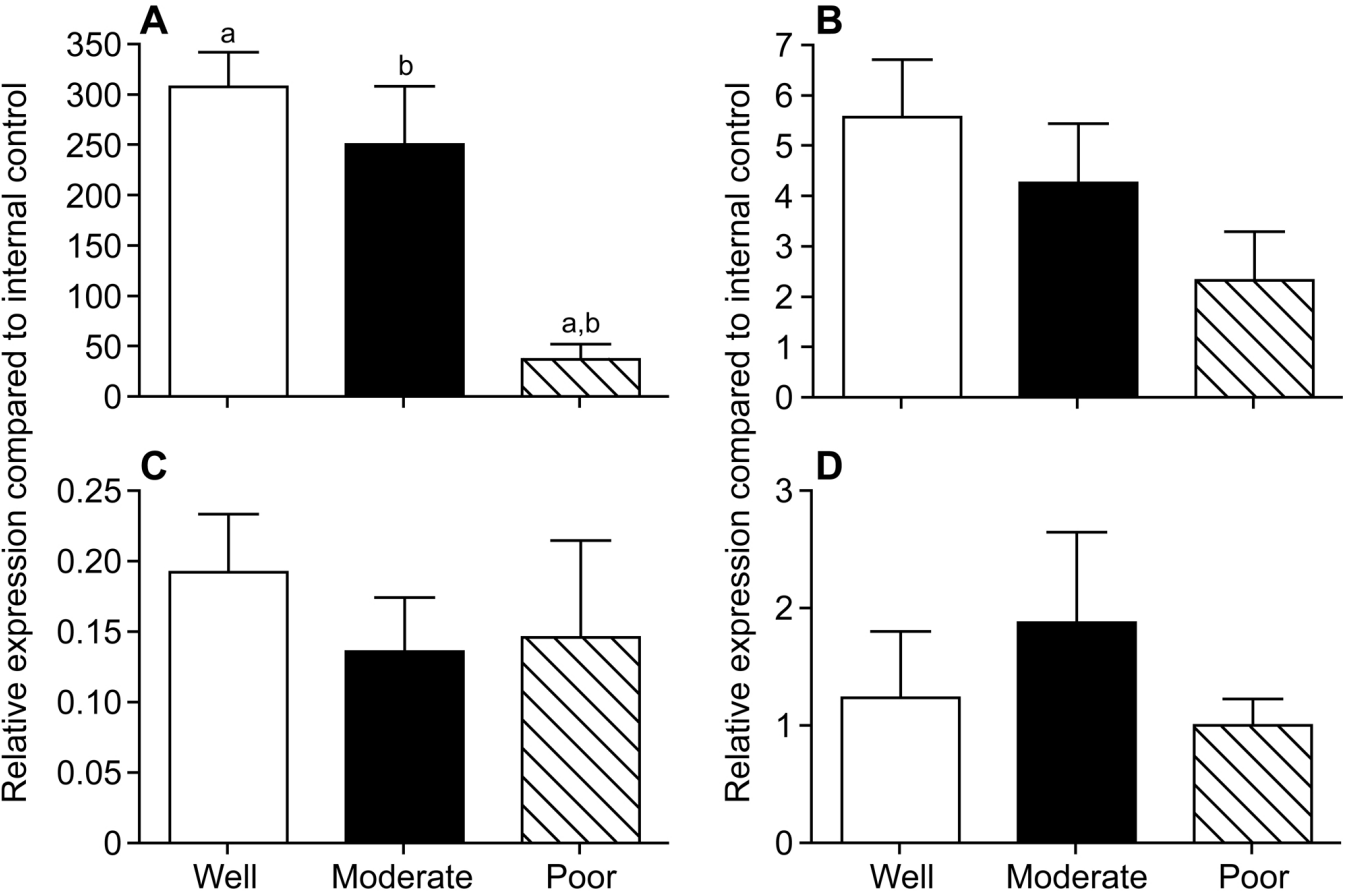
**Expression of mRNAs for ERα, ERβ1 (wild type) and two truncated ERβ variants (ERβ2 and ERβ5) in grade 1 endometrial cancers**. Samples n = 10 per group, concentrations were normalised against those of an internal control sample. A, ERα, note that the amount of mRNA was significantly lower (p < 0.0001) in samples graded as poor compared with those that were well or moderately differentiated; B, ERβ1 (full length wild-type); C, ERβ2 (ERβcx); D, ERβ5. There was no statistically significant difference between the levels of expression of ERβ variants between the different groups. Significant differences between samples are indicated with letters a, b.

The amount of ERα mRNA was significantly lower in poorly differentiated cancers compared with cancers graded as well or moderately differentiated (Figure 1A). Messenger RNAs for ERβ1 (Figure 1B), ERβ2 (Figure 1C) and ERβ5 (Figure 1D) did not vary significantly with grade although there was a trend for a reduction in the total amount of ERβ1 mRNA in the poorly differentiated cancers.

**Figure 2:**
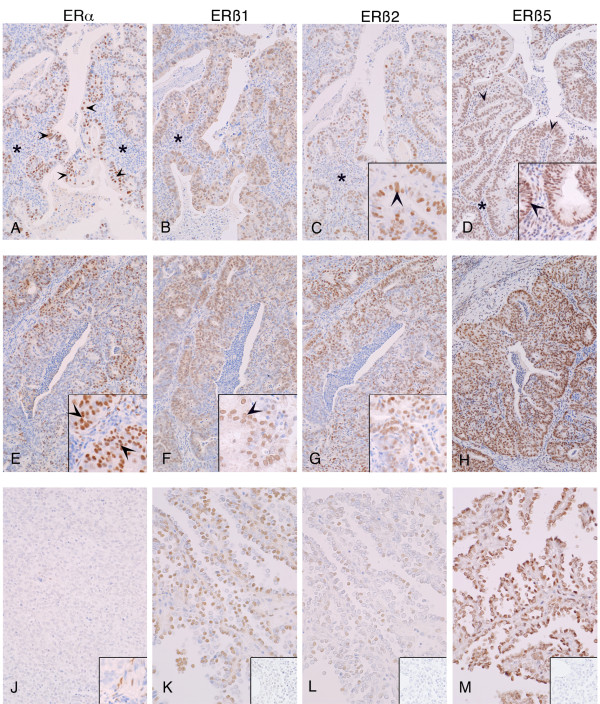
**Immunoexpression of ERs in endometrial cancers**. Tissues were classified as well (A-D), moderately (E-H) or poorly (J-M) differentiated; main panels show closely adjacent sections from three cancer blocks to allow direct comparisons. All proteins were immunolocalised to cell nuclei (see higher power inserts in panels e, f, c and d respectively). In the well and moderately differentiated cancers expression was most intense in epithelial cell layers (arrowheads, panel A and inserts). Note that ERα was low/absent in poor grade cancers (J) but immunoexpression of ERβ1, 2, 5 was readily detected (K. L, M). Inserts in panels K, L, and M show negative controls for ERβ1, ERβ2 and ERβ5 antibodies respectively generated using primary antibodies pre-absorbed with specific peptides used for immunisation. Asterisks (*) label the stromal compartment that was well defined in the well differentiated cancers.

Expression of ERβ isoforms (ERβ1, β2 and β5) was detected using variant-specific monoclonal antibodies, All three proteins were immunolocalised to cell nuclei and in the well and moderately differentiated tissues positive staining was detected in both epithelial and stromal compartments (Figure 2B, C, D, F, G, H). Immunoexpression of ERβ5 was intense in most samples regardless of grade (Figure 2D, H, M). Consistent with previous reports immunoexpression of ERα was intense in epithelial cell nuclei in well and moderately differentiated cancers (Figure 2A, C arrowheads in panel A and at higher power in the inset of panel E) but little or no protein was detected in the poorly differentiated cancers (Figure 2J). The inset in Figure 2 panel J illustrates some of the few immunopositive cells detected in the poorly differentiated samples all of which appeared to have a stromal/fibroblast phenotype.

### Expression of PR was down regulated in the poorly differentiated cancers and paralleled expression of ERα

**Figure 3:**
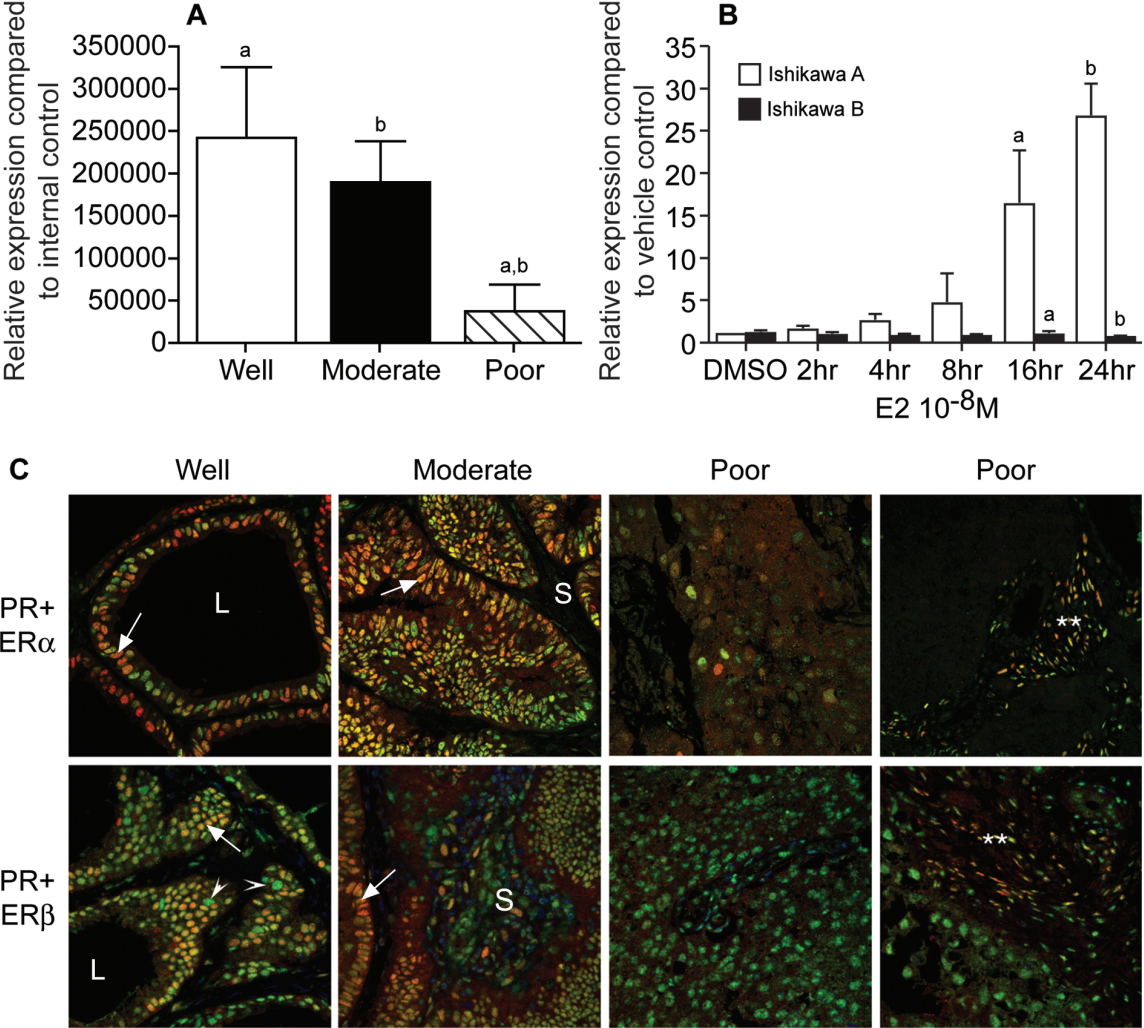
**Expression of PR paralleled that of ERα not ERβ**. A. Expression of PR mRNA was significantly higher in cancers that were classified as well or moderately differentiated as compared with those with a poorly differentiated phenotype (p < 0.05, n = 10 cancers in each group, comparisons indicated by letters a, b). B. PR mRNA was significantly higher in Ishikawa A [ERα-positive] compared to Ishikawa B [ERα-negative] after incubation with E2 for 16 (a) or 24 (b) hours (p < 0.01). Values are expressed as mean +/- SD of three independent experiments performed in duplicate. C. Fluorescent co-localisation as carried out using antibodies specific for ERα or ERβ1 (both green) and PR (red). The cancers illustrated were classified as well (code 1614), moderately (code 1930) or poorly (c, codes 0001 and 1176) differentiated; at least 8 samples were analysed in each group. Co-expression was detected as yellow/orange immunofluoresence. In the well and moderately differentiated cancers expression of PR was most intense in epithelial cells and broadly overlapped with that of ERα (e.g. in cells indicated by arrows). Expression of PR was very low in the poorly differentiated cancers and appeared confined to cells with a fibroblast phenotype (**). Some ERβ1 positive cells were PR positive however most cells in the poorly differentiated cancers were ERβ1 positive and PR negative (green nuclei). Labels: L = lumen, S = stromal compartment, arrowheads = ERβ1 postive cells that are PR negative.

The amount of PR mRNA in endometrial cancer homogenates was grade dependent and was significantly lower in the poorly differentiated cancers compared to those classified as well or moderately differentiated (Figure 3A). Incubation of ERα-positive Ishikawa (A) cells with 10^-8^M E2 resulted in a significant, time dependent, increase in expression of PR mRNA reaching ~25 fold above controls at 24 h however there was no detectable increase in expression of PR mRNA in ERα-negative Ishikawa cells (Figure 3B). Both Ishikawa cell lines expressed mRNAs for ERβ1, ERβ2 and ERβ5 with higher concentrations in the ERα-negative cells [see Additional file 1]. The presence of functional ERs was confirmed using a luciferase reporter transgene driven by a 3xERE response element [see Additional file 1]; as expected in ERα-positive cells expression of luciferase was induced by E2; no increase in reporter gene activity was detected in the ERα-negative cells unless the cells were infected with an adenoviral construct containing an ERα cDNA. These results demonstrate that although the cells contain all the factors essential for induction of ERE-dependent gene expression this could not be induced by ligand-activation of ERβ1.

Fluorescent immunohistochemistry revealed that PR was widely expressed in the nuclei of epithelial cells in both well and moderately differentiated cancers but most cells in the poorly differentiated samples were immunonegative (Figure 3C). Most, but not all, of the epithelial cells within the well and moderately differentiated cancers co-expressed both PR and ERα (yellow/orange nuclei, Figure 3C). In the poorly differentiated cancers very few cells were immunopositive for PR and most of these were ERα-positive with a fibroblastic phenotype (Figure 3C, labelled ** in upper right panel). Co-immunostaining for PR and ERβ1 identified cells that were ERβ1 positive/PR negative in the epithelial layer of the well differentiated cancers (e.g. arrowheads lower left panel). In the poorly differentiated cancers the majority of cells that were immunopositive for ERβ1 did not express PR (green nuclei) although the population of fibroblastic cells identified in the same samples stained with ERα (see above) were PR positive and a few co-expressed ERβ1 (yellow nuclei, ** Figure 3C lower right).

### Expression of COX-2 in epithelial cells is associated with reduced expression of ERα

**Figure 4:**
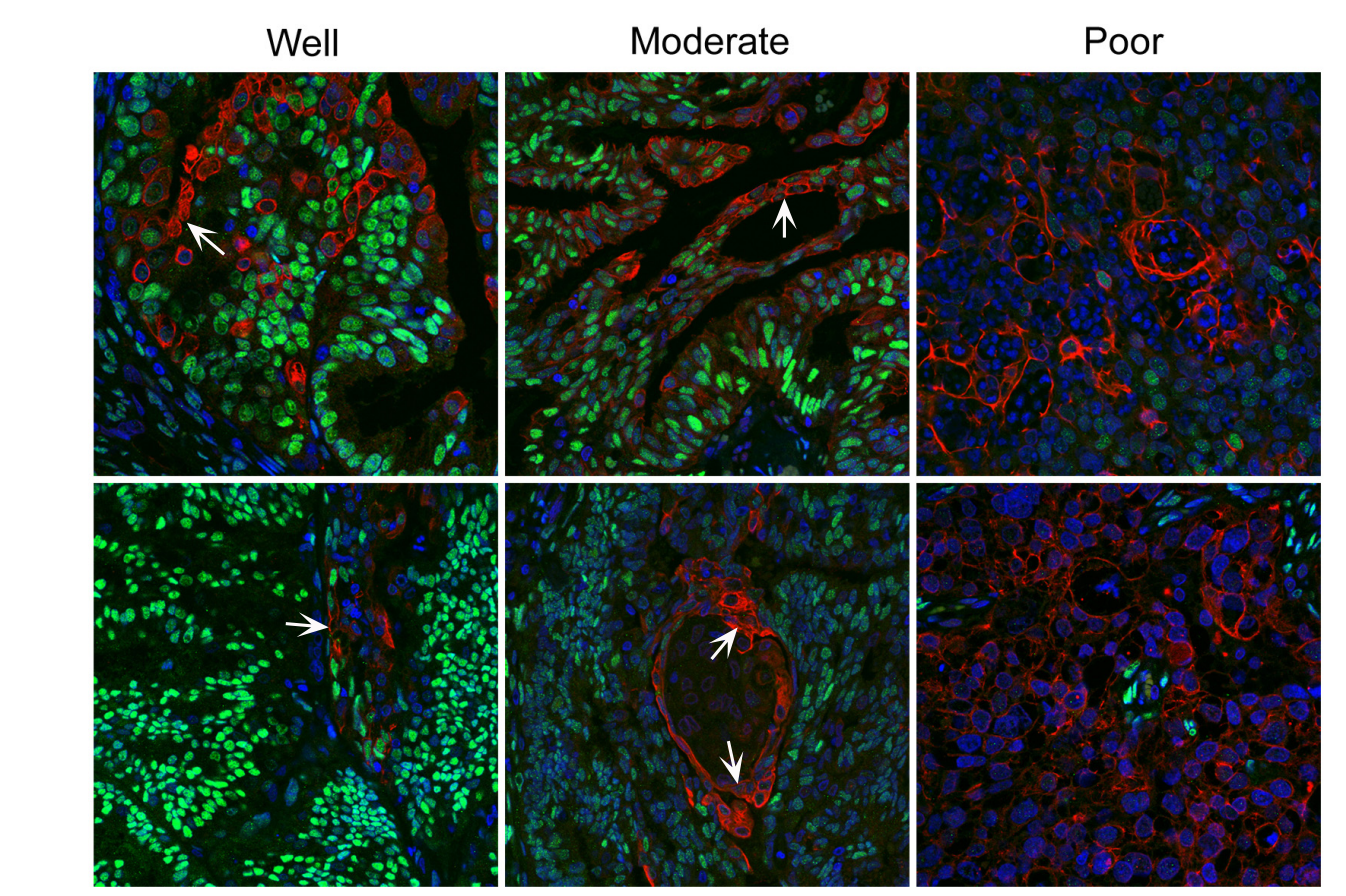
**Double immunohistochemistry for COX-2 (red) ERα (green)**. Immunoexpression of COX-2 was confined to the cytoplasm; a greater proportion of the cells were immunopositive in the poorly differentiated tissue than in well or moderately differentiated samples. Immunoexpression of COX-2 and ERα appeared to be inversely related e.g. arrows COX-2^pos^/ERα^neg ^cells.

Immunoexpression of COX-2 was localised specifically to the cytoplasm of epithelial cells in the well (n = 10) and moderately (n = 9) differentiated cancers (Figure 4 red staining). In well and moderately differentiated samples the amount of ERα in cell nuclei generally appeared to be lower in the COX-2 positive cells (arrows) than in the surrounding tissue (intense green staining of ERα-positive cell nuclei) and in the poorly differentiated samples nearly all the COX-2 positive cells were ERα-negative prompting us to use a model cell line to explore whether treatment of cells with prostaglandin F could have an impact on expression of ERα. PGF2α was used in these studies as our previous work had shown that this prostaglandin is synthesised in endometrial adenocarcinomas [41] and can drive epithelial cell proliferation in endometrial tissue [32].

### Incubation of Ishikawa cells with prostaglandins alters expression of ERα and PR

**Figure 5:**
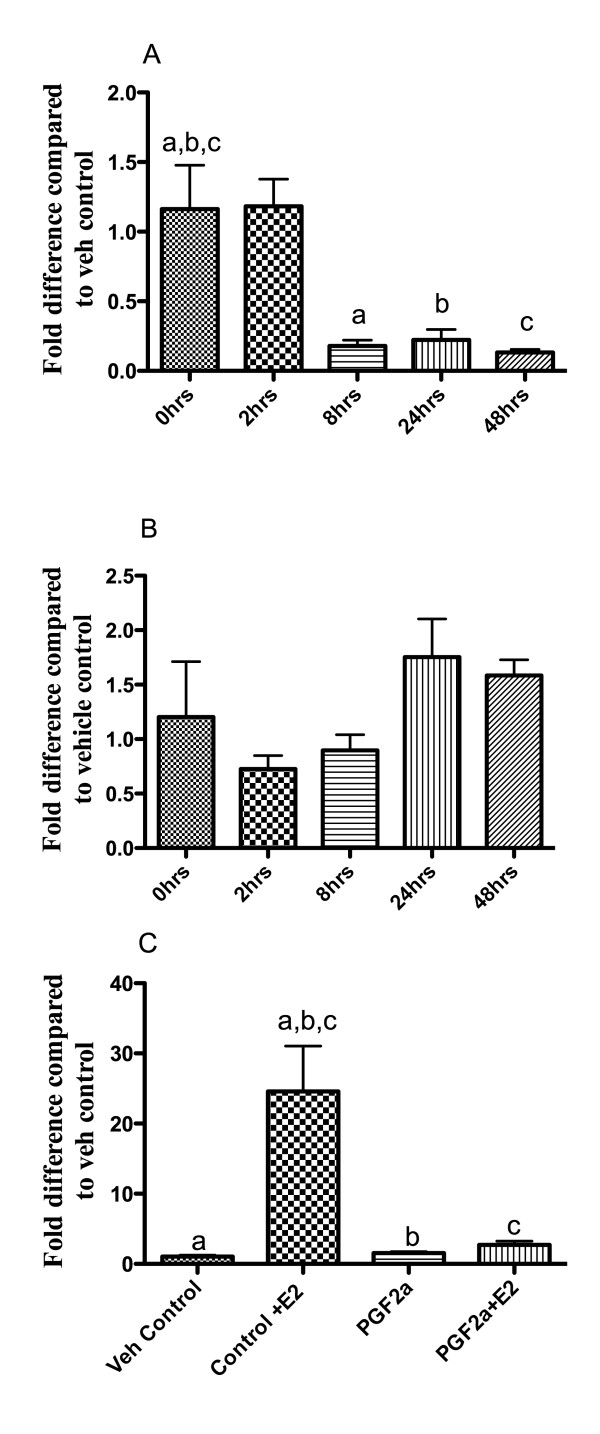
**Cell based studies suggest that local biosynthesis of prostaglandin could regulate expression of ERα and PR**. Incubation of Ishikawa cells with PGF2α resulted in reduced expression of ERα mRNA. Panel A, Samples which differed significantly from each other are indicated by letters a, b, c; p < 0.005 in all cases. Panel B, there was no significant impact on expression of ERβ1 mRNA between samples at the start or end of the experiment. Panel C, Samples which differed significantly from each other are indicated by letters a, b, c; p < 0.001 in all cases. Incubation of cells with E2 for 24 h resulted in a significant increase in expression of PR mRNA this rise did not occur if cells were pre-incubated with PGF2α for 24 h. All values are expressed as mean +/- SD of three independent experiments performed in duplicate.

Incubation of Ishikawa cells expressing both ERα and the FP receptor [31] with PGF2α resulted in a significant (p < 0.005) and sustained down-regulation in expression of ERα mRNA (Figure 5A) but no significant change in the amount of ERβ1 mRNA (Figure 5B). In a follow up study the ability of cells to up-regulate expression of PR mRNA in response to treatment with E2 (10^-8^M, 24 hours) was investigated in control cells and those pre-incubated with ERα for 24 hours. In line with expectations incubation of control cells with E2 for 24 hours resulted in increased expression of PR mRNA however pre-incubation with PGF2α significantly blunted the response to E2 treatment (p < 0.001) a finding consistent with the reduction in expression of ERα as a result of PGF2α treatment.

## Discussion

A recent paper reported that women with variants of the aromatase (*CYP19A1*) gene that are associated with a 10-20% increase in circulating oestrogen levels after menopause have an increased risk of endometrial cancer [42], In the present study we have examined the patterns of expression of ERα, the full length ERβ receptor (ERβ1) and two ERβ splice variant isoforms (ERβ2, ERβ5) in well-characterised stage I endometrioid adenocarcinomas. This extends a preliminary study that discovered ERβ2 and ERβ5 mRNAs were more abundant than those of ERβ4 in human endometrium and Ishikawa cells [43].

In a fixed tissue set comprising 30 well characterised cancers (well, moderately and poorly differentiated) expression of ERα was reduced in the poorly differentiated tissues a finding that is in agreement with previous reports [14,44]. Although studies in rodents have demonstrated that ERα-dependent gene activation plays a key role in endometrial epithelial cell proliferation [45] in our samples proliferative activity of endometrial adenomas (as determined by immunostaining for Ki67 or histone H3, unpublished observations) was highest in the poorly differentiated tumours even when they were ERα-negative (not shown). These results agree with a recent study documenting increased expression of Ki67 and other cell cycle regulators such as cyclin A during the progression from a normal to malignant endometrial phenotype [46] and higher expression of Ki67 in ERα-negative tumours with a more aggressive phenotype [47].

To date studies on the role(s) played by ERβ in disease progression, cell survival and proliferation have been dominated by studies on breast cancer tissues and breast cancer cell lines. In these samples over-expression of ERβ results in anti-proliferative and pro-apoptotic effects [48] and expression of ERβ2 correlates with favourable response to endocrine therapy and improved survival [49]. Other studies have reported no correlation between expression of ERβ2 mRNA and response to tamoxifen [50,51]. A recent study used tissue microarrays to determine expression of ERβ1, β2 and β5 in a series of 880 cases of primary invasive breast carcinomas from patients with long term follow up. Expression of ERβ2 or ERβ5, but not ERβ1 significantly correlated with overall survival [39]. To date only two studies have examined expression of ERβ in endometrial cancers. In both studies samples were ERα-positive; one group reported detection of ERβ5 mRNA [16] the other reported finding no correlation between ERβ mRNA expression and PR labeling index, cell proliferation or histologic grade [15]. We believe this is the first paper demonstrating immunoexpression of ERβ5 protein in cell nuclei within stage 1 endometrial adenocarcinomas regardless of whether they were well or poorly differentiated. Expression of ERβ5 is not unique to tumour cells and we have immunolocalised the protein to multiple cell types in normal cycling endometrium, first trimester decidua and placenta (Fitzgerald, MacPherson and Saunders, unpublished observations). Molecular modelling of the ERβ5 protein suggests that it does not contain a functional ligand-binding pocket [10]. ERβ5 has been demonstrated to form a hetero-dimeric complex with ERα which negatively regulated transcriptional activity [52]: this may explain why ERβ5 expression was associated with a better prognosis in breast cancer [53]. Leung et al [10] detected increased activation of an ERE-luciferase reporter in HEK293 cells incubated with oestrogens including E2 when cells were co-transfected with ERβ1 and ERβ5 compared with those transfected with ERβ1 alone.

In the current study expression of PR in endometrial adenocarcinoma tissues broadly paralleled that of ERα with minimal expression of PR in the poorly differentiated cancers even though these tissues maintained expression of ERβ. In our ERα^pos^/ERβ^pos ^Ishikawa (A) cells expression of PR mRNA and a luciferase gene driven by a consensus 3xERE promoter were both induced by E2 treatment. No activity was detected in the ERα^neg^/ERβ^pos ^Ishikawa cells (line B) even though they were able to activate the ERE-luciferase when ERα was reintroduced into the cells suggesting the lack of response was not due to lack of transcriptional competence; both cell lines expressed similar concentrations of ERβ5 mRNA. Our results are in agreement with those of others [54] who reported that ERβ was unable to up-regulate expression of the PRB promoter in HeLa, BT-20 or Ishikawa cells although in SK-BR-3 cells both receptors were able to repress promoter activity. The potential that ERβ-dependent gene activation can occur in the endometrial cancers is supported by the results of studies using tamoxifen, a SERM that acts as a potent transcriptional activator of ERβ at AP-1 response elements [55]. Treatment with tamoxifen results in a more aggressive endometrial cancer phenotype and development of a distinctive 'tamoxifen-specific' gene profile [56,57].

Expression of COX-2 but not COX-1 is up-regulated in endometrial adenocarcinoma compared with expression levels observed in normal endometrium [27,28]. This is associated with increased biosynthesis of prostaglandins and increased expression of FP receptors resulting in a stimulation of FP-receptor dependent signalling and production of angiogenic factors. [29]. In addition there is evidence that PGE2 can up-regulate expression of steroidogenic genes including *CYP19A1 *and thereby contribute to increased local concentrations of oestrogenic ligands that could bind ERα and/or ERβ [58]. We believe the data in the present paper provide preliminary evidence for a link between signalling via the FP receptor and an apparent reduction in expression of ERα and PR. The human ERα gene is transcribed from at least seven promoters into multiple transcripts that vary in their 5' UTRs. Tissue specific expression of transcripts has been documented as having differential use of promoters in normal and cancerous breast tissue (reviewed in [59]). The signalling pathway responsible for down regulation in the amount of ERα mRNA after incubation of endometrial Ishikawa cells with prostaglandin F2α requires further investigation in order to determine whether the effects we observed are mediated by transcriptional or post transcriptional mechanism(s).

## Conclusion

Our results shed new light on the interplay between PG and ER-dependent patterns of gene expression in endometrial cancers. First we would speculate that ligand-dependent or ligand-independent activation of ERβ isoforms could have an impact on progression of endometrial cancers especially those with a more aggressive phenotype that are ERα-negative and this merits further investigation. Second, although increased biosynthesis of prostaglandins is known to occur in endometrial cancers we believe our study provides the first evidence that down-regulation in expression of ERα, and the consequent reduction in expression of PR, may be one of the downstream consequences of F prostaglandin-dependent signalling.

## Competing interests

The authors declare that they have no competing interests.

## Authors' contributions

FC carried out studies using cell cultures and performed QRTPCR and reporter assays. SM performed the immunohistochemistry. VB and PB cloned and prepared viral constructs. RAA collected the tissues; ARWW examined sections of tumours and graded them. PTKS and HJN initiated the study and designed the experiments. All authors contributed to the preparation of the final manuscript.

## Pre-publication history

The pre-publication history for this paper can be accessed here:

http://www.biomedcentral.com/1471-2407/9/330/prepub

## Supplementary Material

Additional file 1 **Expression of ERα and ERβ in two adenocarcinoma-derived Ishikawa cell lines mirrors that of well and poorly differentiated cancers**. This figure shows analysis of expression of ER mRNAs and E2 responsiveness of the two Ishikawa cell lines used in the study. Messenger RNAs detected by qRTPCR: A, ERα; B, ERβ1; C, ERβ2; D, ERβ5. Note that Ishikawa A (white bars) were characterised as having abundant ERα whereas expression of ERα in Ishikawa B cells (black bars) was minimal. In contrast, expression of ERβ mRNA was higher in Ishikawa B than Ishikawa A and all three splice variant isoforms were expressed (ERβ1, ERβ2 and ERβ5). E. Ishikawa A cells incubated with 10^-10 ^to 10^-7 ^M E2 were able to induce expression of a luciferase construct under the control of a 3xERE promoter whereas no expression was noted when Ishikawa B cells were infected with the same construct and incubated under identical conditions. Induction of the ERE-luciferase reporter in Ishikawa B cells in response to treatment with E2 was restored by introduction of an ERα cDNA (grey bars). Click here for file
