# Supplementary figures and images for: Expression of oestrogen receptors, ERα, ERβ, and ERβ variants, in endometrial cancers and evidence that prostaglandin F may play a role in regulating expression of ERα
Source: BMC Cancer. 2009 Sep 16;9:330. doi: 10.1186/1471-2407-9-330 (PMC2755482; doi:10.1186/1471-2407-9-330)

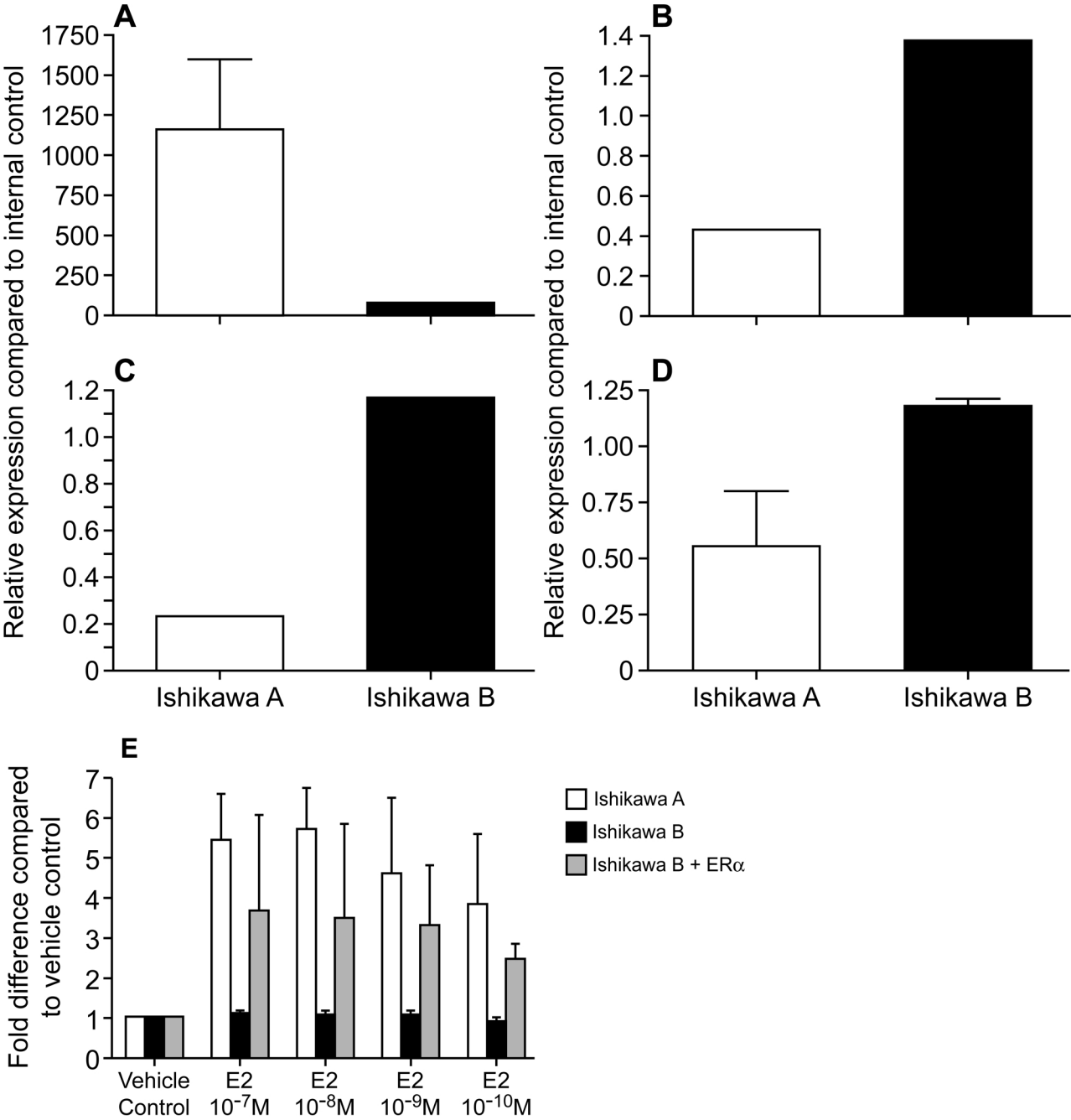

Supplement: Additional file 1 — Expression of ERα and ERβ in two adenocarcinoma-derived Ishikawa cell lines mirrors that of well and poorly differentiated cancers. This figure shows analysis of expression of ER mRNAs and E2 responsiveness of the two Ishikawa cell lines used in the study. Messenger RNAs detected by qRTPCR: A, ERα; B, ERβ1; C, ERβ2; D, ERβ5. Note that Ishikawa A (white bars) were characterised as having abundant ERα whereas expression of ERα in Ishikawa B cells (black bars) was minimal. In contrast, expression of ERβ mRNA was higher in Ishikawa B than Ishikawa A and all three splice variant isoforms were expressed (ERβ1, ERβ2 and ERβ5). E. Ishikawa A cells incubated with 10-10 to 10-7 M E2 were able to induce expression of a luciferase construct under the control of a 3xERE promoter whereas no expression was noted when Ishikawa B cells were infected with the same construct and incubated under identical conditions. Induction of the ERE-luciferase reporter in Ishikawa B cells in response to treatment with E2 was restored by introduction of an ERα cDNA (grey bars). [file 1471-2407-9-330-S1.pdf]
